# Fulminant Ischemic Colitis With Toxic Megacolon in a Young Man Receiving Testosterone Replacement Therapy Despite Therapeutic Phlebotomy for Hereditary Hemochromatosis: A Case Report

**DOI:** 10.7759/cureus.116752

**Published:** 2026-09-22

**Authors:** Ahed Anbari, Leslie Caren

**Affiliations:** 1 Internal Medicine and Family Medicine, Trinity Health Oakland Hospital, Pontiac, USA; 2 Internal Medicine, Trinity Health Oakland Hospital, Pontiac, USA

**Keywords:** fulminant ischemic colitis, hereditary hemochromatosis, non occlusive mesenteric ischemia, persistent erythrocytosis, relative hemoconcentration, subtotal colectomy with ileostomy, testosterone replacement therapy, therapeutic phlebotomy, toxic megacolon

## Abstract

Fulminant ischemic colitis with toxic megacolon requiring emergent colectomy is uncommon in younger adults and warrants evaluation for unusual etiologies and potentially modifiable risk factors. Testosterone replacement therapy (TRT) is a recognized cause of secondary erythrocytosis, while acute volume depletion and systemic hypoperfusion may further compromise mesenteric perfusion.

We report the case of a 43-year-old man with hereditary hemochromatosis managed with therapeutic phlebotomy, hypogonadism treated with intramuscular testosterone, type 2 diabetes mellitus, stage 3a chronic kidney disease, hyperlipidemia, obesity, and active tobacco use who presented with three days of progressive abdominal distention, markedly decreased oral intake, severe abdominal pain, and syncope. Laboratory evaluation demonstrated erythrocytosis, with a hemoglobin of 17.9 g/dL and hematocrit of 52.2% despite therapeutic phlebotomy 13 days earlier, along with severe hyperglycemia and renal dysfunction in a clinical context supportive of volume depletion. Computed tomography demonstrated diffuse colonic dilation and pneumatosis involving the ascending colon. Emergent exploratory laparotomy revealed toxic megacolon with extensive necrotic changes extending from the cecum to the proximal descending colon of unclear etiology, requiring subtotal colectomy with end ileostomy. Surgical pathology demonstrated mucosal hemorrhage and focal transmural acute inflammation, with viable resection margins and no evidence of malignancy. An extensive thrombophilia evaluation was unrevealing, including testing for Factor V Leiden, prothrombin G20210A mutation, antiphospholipid antibodies, lupus anticoagulant, antithrombin deficiency, and protein S deficiency. Following discharge, erythrocytosis persisted despite recent phlebotomy, with hemoglobin 17.1 g/dL and hematocrit 51%.

This case highlights a potentially multifactorial pathway to severe colonic ischemia involving persistent erythrocytosis during testosterone therapy, acute volume depletion, severe colonic distention, and underlying vascular risk factors. Although causality between TRT-associated erythrocytosis and colonic ischemia cannot be established, the combination may have contributed to impaired microvascular perfusion in a susceptible patient.

The case emphasizes the importance of monitoring hematocrit in patients receiving testosterone therapy, particularly when additional risk factors for hypoperfusion or thrombosis are present, and highlights the importance of coordinated postoperative care to ensure timely access to essential ostomy supplies.

## Introduction

Ischemic colitis is the most common form of intestinal ischemia and typically affects older adults, particularly those with atherosclerotic disease [1]. Fulminant ischemic colitis progressing to toxic megacolon and requiring emergent colectomy is uncommon in younger adults and should prompt evaluation for inherited thrombophilia, acquired hypercoagulable conditions, medication-related etiologies, hypoperfusion, and other vascular risk factors [2,3]. Toxic megacolon is most commonly associated with inflammatory bowel disease or infectious colitis, although other etiologies have also been reported [4,5]. In the absence of an occlusive vascular lesion, nonocclusive low-flow mechanisms should also be considered [6]. Testosterone replacement therapy (TRT) is widely used in the treatment of male hypogonadism, and erythrocytosis is a recognized adverse effect requiring hematocrit monitoring during treatment [7,8]. Testosterone can stimulate erythropoiesis through increased erythropoietin signaling and suppression of hepcidin [9,10].

We report a case of fulminant ischemic colitis with toxic megacolon in a young patient receiving TRT despite routine therapeutic phlebotomy for hereditary hemochromatosis.

## Case presentation

A 43-year-old man with hereditary hemochromatosis managed with therapeutic phlebotomy every three months, hypogonadism treated with intramuscular testosterone cypionate, type 2 diabetes mellitus complicated by stage 3a chronic kidney disease, hyperlipidemia, obesity (body mass index 30.8 kg/m²), generalized anxiety disorder, depression, and active tobacco use presented with progressively worsening abdominal distention and severe abdominal pain.

Three days before presentation, he developed progressive abdominal bloating accompanied by markedly decreased oral intake because of increasing abdominal discomfort and distention. During this period, he reported consuming minimal food and fluids. On the day of admission, he experienced severe diffuse abdominal pain followed by a syncopal episode while exiting his mother's vehicle en route to the Emergency Department. His most recent therapeutic phlebotomy for hereditary hemochromatosis had been performed approximately two weeks before presentation.

Initial laboratory evaluation revealed persistent erythrocytosis with a hemoglobin level of 17.9 g/dL and hematocrit of 52.2%, despite recent therapeutic phlebotomy. Additional laboratory findings included severe hyperglycemia (glucose 518 mg/dL), hyponatremia (129 mmol/L), acute kidney injury (creatinine 1.8 mg/dL), mildly elevated lipase (163 U/L), and a lactate level of 2.2 mmol/L. Collectively, these findings were consistent with significant dehydration, severe hyperglycemia, and acute kidney injury. Other hematologic indices, liver function tests, coagulation studies, and serum proteins were within normal limits or demonstrated no clinically significant abnormalities. Pertinent laboratory findings are summarized in Table 1.

**Table 1:** Pertinent admission laboratory findings *Reference range may vary by laboratory

Laboratory Test	Patient Value	Reference Range	Clinical Interpretation
Hemoglobin	17.9 g/dL	13.5-17.5 g/dL	Persistent erythrocytosis despite recent therapeutic phlebotomy
Hematocrit	52.20%	41.0-53.0%	High-normal
RBC count	6.41 × 10⁶/µL	4.50-5.90 × 10⁶/µL	Consistent with erythrocytosis
White blood cell count	10.31 × 10³/µL	4.0-11.0 × 10³/µL	Within reference range
Platelet count	198 × 10³/µL	150-400 × 10³/µL	Normal
Sodium	129 mmol/L	135-145 mmol/L	Hyponatremia in setting of severe hyperglycemia
Chloride	95 mmol/L	98-107 mmol/L	Mild hypochloremia
CO₂ (Bicarbonate)	20.1 mmol/L	22-29 mmol/L	Mild metabolic acidosis
Anion Gap	13.9 mmol/L	3-11 mmol/L	Mildly elevated
Creatinine	1.8 mg/dL	0.7-1.3 mg/dL	Acute kidney injury
eGFR	47 mL/min/1.73 m²	>60 mL/min/1.73 m²	Reduced renal function
Glucose	518 mg/dL	70-99 mg/dL	Severe hyperglycemia
Lactate	2.2 mmol/L	0.5-2.0 mmol/L	Mildly elevated
Lipase	163 U/L	13-60 U/L*	Mild elevation without clinical pancreatitis
High-Sensitivity Troponin T	11 ng/L	<14 ng/L	Within reference range
Prothrombin Time	11.3 seconds	11-13.5 seconds	Normal
INR	1.01	0.8-1.2	Normal

Contrast-enhanced computed tomography (CT) of the abdomen and pelvis, obtained from an external CT report, demonstrated diffuse gaseous distention of the colon without a definite transition point, tiny foci of pneumatosis involving the ascending colon, and marked gastric distention with retained fluid extending into the distal esophagus. The imaging findings raised concern for ischemic colitis, although infectious colitis, inflammatory colitis, and idiopathic pneumatosis remained within the differential diagnosis. Representative sagittal, coronal, and axial CT images are shown in Figure 1.

**Figure 1:**
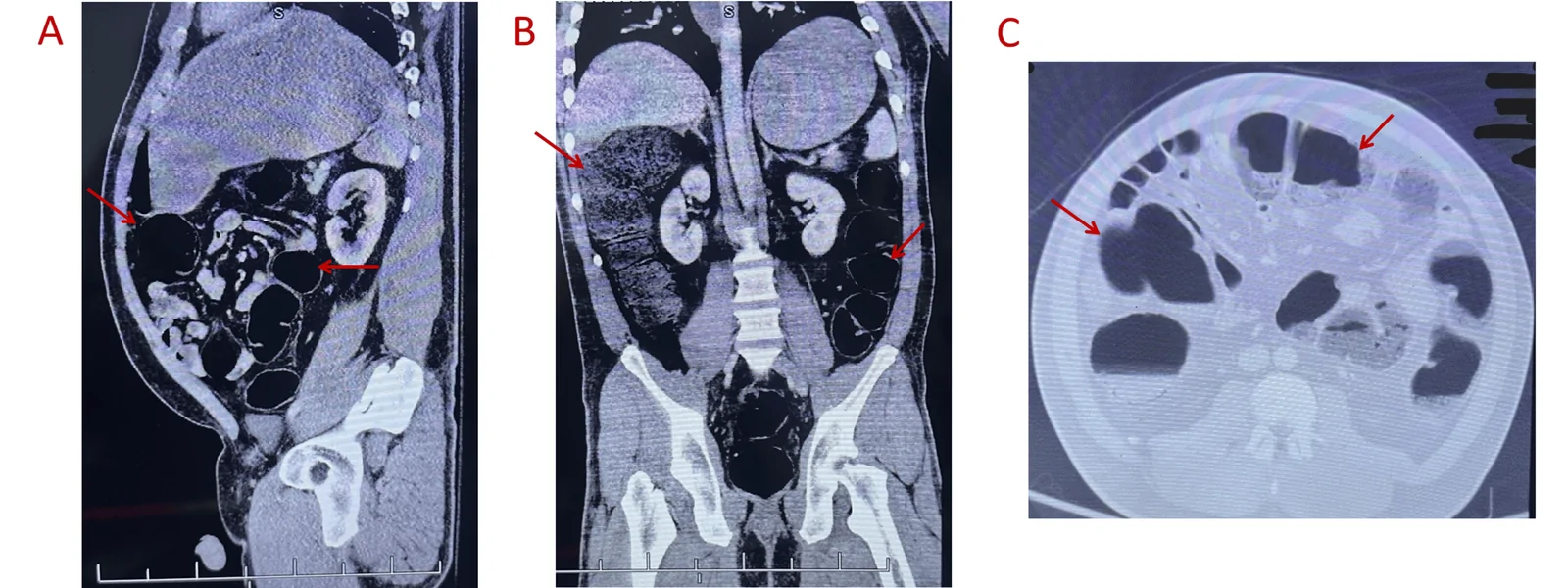
Representative contrast-enhanced CT images of the abdomen and pelvis (A) Sagittal image demonstrating multiple dilated gas-filled bowel loops and marked abdominal distention. (B) Coronal image demonstrating extensive colonic dilation without a definite transition point. (C) Axial image demonstrating diffuse colonic gaseous distention.

Because of worsening abdominal distention and concern for bowel ischemia, the patient underwent emergent exploratory laparotomy. Intraoperatively, extensive necrotic changes involving the colon from the cecum to the proximal descending colon were identified in the setting of toxic megacolon. No definitive etiology for the necrosis was identified during surgery. Approximately 80% of the colon was resected, and an end ileostomy was created.

Histopathologic examination of the resected specimen demonstrated colonic mucosal hemorrhage with focal transmural acute inflammation. Both proximal and distal surgical margins were viable, and there was no evidence of malignancy, chronic inflammatory bowel disease, or other underlying pathologic process to explain the extensive colonic necrosis.

Given the patient's relatively young age and persistent erythrocytosis, an extensive evaluation for inherited and acquired thrombophilia was performed following surgery. Testing for Factor V Leiden mutation, prothrombin G20210A mutation, lupus anticoagulant, anticardiolipin antibodies, beta-2 glycoprotein I antibodies, antithrombin III deficiency, and protein S deficiency was negative. Protein C activity was mildly elevated at 142%, a finding not considered clinically significant. Results of the hypercoagulable evaluation are summarized in Table 2. Follow-up laboratory testing approximately two weeks after discharge continued to demonstrate persistent erythrocytosis, with a hemoglobin level of 17.1 g/dL and hematocrit of 51%, despite therapeutic phlebotomy performed shortly before hospitalization.

**Table 2:** Hypercoagulable evaluation

Test	Result	Interpretation
Factor V Leiden mutation	Negative	No inherited thrombophilia
Prothrombin G20210A mutation	Negative	No inherited thrombophilia
Lupus anticoagulant	Negative	No evidence of antiphospholipid syndrome
Anticardiolipin IgG	Negative	Normal
Anticardiolipin IgM	Negative	Normal
Anticardiolipin IgA	Negative	Normal
β2-Glycoprotein I IgG	Negative	Normal
β2-Glycoprotein I IgM	Negative	Normal
β2-Glycoprotein I IgA	Negative	Normal
Antithrombin III activity	93%	Normal
Protein S activity	105%	Normal
Protein C activity	142%	Mildly elevated; not considered clinically significant

The patient recovered without recurrent bowel ischemia. However, his postoperative course was complicated by delays in obtaining ileostomy appliances, skin barriers, barrier rings, and home health services after discharge. These issues required extensive coordination among the colorectal surgery team, primary care physician, home health agency, and durable medical equipment providers before appropriate outpatient ostomy management could be established.

## Discussion

Ischemic colitis is the most common form of gastrointestinal ischemia and generally results from transient or sustained reduction in colonic blood flow. Although it is more frequently encountered in older adults with atherosclerotic disease, ischemic colitis in younger patients should prompt consideration of nontraditional risk factors, including hypercoagulable states, medications, vasculitis, hypoperfusion, and other conditions affecting mesenteric blood flow. The pathophysiology is often multifactorial rather than attributable to a single vascular event [1-3]. In this case, the development of extensive colonic necrosis and toxic megacolon in a 43-year-old man without an identified arterial or venous occlusion raises the possibility of a multifactorial hypoperfusion mechanism.

The severity and distribution of disease in this patient were notable. CT demonstrated diffuse colonic gaseous distention with pneumatosis involving the ascending colon, while operative exploration subsequently demonstrated toxic megacolon with necrotic changes extending from the cecum to the proximal descending colon. Approximately 80% of the colon required resection. Histopathologic examination demonstrated mucosal hemorrhage and focal transmural acute inflammation with viable surgical margins and no evidence of malignancy or chronic inflammatory bowel disease. Toxic megacolon is most commonly associated with inflammatory bowel disease or infectious colitis, although other inflammatory and ischemic conditions have also been reported as potential precipitants [4,5]. Ischemic colitis is an uncommon but recognized cause of toxic megacolon [4,5]. The absence of chronic inflammatory bowel disease or malignancy in this patient makes the operative finding of toxic megacolon particularly unusual and supports consideration of ischemic injury as an initiating or contributing process.

Evaluation for an underlying hypercoagulable state

Because of the patient's young age and extensive bowel ischemia, an evaluation for acquired and inherited thrombophilia was performed. Testing for Factor V Leiden, prothrombin G20210A mutation, lupus anticoagulant, anticardiolipin antibodies, beta-2 glycoprotein I antibodies, antithrombin III deficiency, and protein S deficiency was unrevealing. Protein C activity was mildly elevated at 142%, which is not considered a clinically meaningful prothrombotic abnormality. These findings make several major inherited and acquired thrombophilic disorders less likely, although they do not completely exclude transient or unrecognized prothrombotic mechanisms.

Importantly, no arterial or venous thrombus was identified on the available imaging or operative evaluation. Therefore, this case should not be characterized as mesenteric thrombosis. Instead, the clinical findings are more compatible with nonocclusive intestinal hypoperfusion occurring in the setting of multiple converging physiologic stressors. Nonocclusive mesenteric ischemia is traditionally associated with reduced systemic perfusion or increased vascular resistance, and ischemic colitis may similarly occur without an identifiable obstructing vascular lesion [2,3,6].

Testosterone-associated erythrocytosis as a potential contributing factor

One of the most clinically relevant findings was persistent erythrocytosis despite ongoing therapeutic phlebotomy for hereditary hemochromatosis. At presentation, the patient's hemoglobin was 17.9 g/dL and hematocrit was 52.2%, with an RBC count of 6.41 × 10⁶/µL. His most recent therapeutic phlebotomy had occurred approximately two weeks before presentation. Follow-up testing after discharge continued to demonstrate erythrocytosis, with a hemoglobin of 17.1 g/dL and hematocrit of 51%.

The patient was receiving intramuscular testosterone cypionate for hypogonadism. Testosterone-associated erythrocytosis is a well-established adverse effect of TRT [7-9]. Testosterone promotes erythropoiesis through several mechanisms, including increased erythropoietin signaling, suppression of hepcidin, and increased iron availability for erythropoiesis [9,10]. Testosterone administration can produce dose-dependent increases in hemoglobin and hematocrit [7,8,11]. Short-acting injectable testosterone formulations are particularly associated with erythrocytosis [7,8,12].

The relationship between testosterone-induced erythrocytosis and thrombosis, however, remains uncertain. Current guidelines emphasize hematocrit monitoring during testosterone therapy and recommend intervention when hematocrit becomes markedly elevated [7,8]. Observational and mechanistic literature suggests that erythrocytosis may contribute to thrombotic risk, but randomized data have not established that testosterone therapy independently causes arterial or venous thrombosis [7,8,12,13]. A systematic review and meta-analysis of randomized controlled trials found no evidence of increased venous thromboembolism with testosterone therapy, although the authors noted limitations in the available evidence [13].

This distinction is important in interpreting our case. The patient's hematocrit of 52.2% was elevated but remained below the commonly cited 54% threshold at which testosterone-associated erythrocytosis generally prompts intervention [7]. The Endocrine Society guideline specifically recommends monitoring hematocrit during testosterone therapy and recognizes erythrocytosis as an important adverse effect [7]. Thus, the case does not demonstrate that his degree of erythrocytosis independently caused intestinal ischemia. Rather, it raises the possibility that even a moderate increase in red cell mass may have become clinically relevant when superimposed on acute volume depletion and impaired regional perfusion.

Interaction between hereditary hemochromatosis, phlebotomy, and testosterone therapy

The coexistence of hereditary hemochromatosis and testosterone therapy creates an unusual clinical context. Hereditary hemochromatosis is characterized by dysregulated iron metabolism and excessive iron accumulation, while therapeutic phlebotomy is the standard approach to reducing iron stores [14,15]. Testosterone independently suppresses hepcidin and promotes erythropoiesis [9,10]. Therefore, these two conditions exert opposing effects on circulating red-cell mass: therapeutic phlebotomy removes erythrocytes, whereas testosterone stimulates erythropoiesis.

The persistent elevation of hemoglobin and hematocrit despite recent phlebotomy suggests that the patient's erythropoietic drive remained substantial. However, it would be inappropriate to conclude that testosterone directly counteracted his phlebotomy or that hereditary hemochromatosis itself caused the ischemic event. Rather, the combination may have created a physiologic environment in which maintaining a normal hematocrit was more difficult.

This distinction is particularly relevant because phlebotomy in hereditary hemochromatosis is primarily directed toward iron depletion, rather than specifically treating testosterone-induced erythrocytosis [14,15]. Consequently, a patient may remain erythrocytotic despite appropriately managed iron overload. The present case highlights the importance of separately evaluating persistent erythrocytosis in patients receiving testosterone, even when they are already undergoing periodic phlebotomy for an unrelated indication.

Dehydration and relative hemoconcentration

A second major feature of this case was substantial reduction in oral intake during the three days preceding hospitalization. Progressive abdominal distention and pain resulted in minimal consumption of both food and fluids. On presentation, the patient had severe hyperglycemia, hyponatremia, elevated creatinine, low serum bicarbonate, and mild lactate elevation. These findings collectively support significant volume depletion and physiologic stress. Severe hyperglycemia may have further contributed to dehydration through osmotic diuresis.

Volume depletion is an established contributor to intestinal hypoperfusion, particularly in patients with limited vascular reserve [1-3,6]. In the present case, dehydration occurred simultaneously with persistent erythrocytosis. Reduced plasma volume would be expected to increase hematocrit through hemoconcentration, potentially increasing whole-blood viscosity. Although the patient's measured hematocrit was not in the range typically associated with severe hyperviscosity syndromes, the physiologic effect may have been amplified by diabetes, chronic kidney disease, active tobacco use, obesity, and other vascular risk factors.

Importantly, the relationship between dehydration and the patient's ischemia is temporally plausible but cannot be proven. Abdominal distention itself likely contributed to reduced oral intake, meaning that dehydration may have been both a consequence of the evolving abdominal process and a factor that subsequently worsened intestinal perfusion. This creates a potentially important vicious cycle: progressive colonic dysfunction and distention reduced oral intake, volume depletion reduced systemic perfusion, and worsening ischemia may have further impaired colonic motility and integrity.

Colonic distention and a possible watershed mechanism

Another possible mechanism is regional hypoperfusion associated with colonic distention. The colon contains areas with relatively limited collateral blood supply, traditionally described as watershed regions, including the splenic flexure and rectosigmoid junction [16,17]. However, the concept that ischemic colitis occurs exclusively or predominantly in these classic watershed regions has been challenged, and ischemic injury can occur outside these locations [17].

This distinction is particularly relevant to our patient. His necrosis extended from the cecum to the proximal descending colon, rather than being restricted to a single classic watershed region. Therefore, a focal watershed event alone is unlikely to explain the extent of his disease. Instead, the distribution may suggest a more diffuse reduction in colonic perfusion.

Severe colonic distention may have further compromised mural perfusion in this patient. Toxic megacolon is characterized by marked colonic dilation and systemic toxicity [4,5]. In the setting of preexisting hypoperfusion, progressive dilation may have further reduced mural perfusion, although this mechanism remains speculative. In our patient, diffuse colonic dilation preceded the discovery of extensive necrosis, making worsening perfusion in the setting of severe dilation a plausible contributing factor rather than an established causal mechanism.

A multifactorial model of intestinal ischemia

Taken together, the findings support a multifactorial model rather than a single identifiable cause. The patient had persistent erythrocytosis while receiving injectable testosterone, despite recent therapeutic phlebotomy. He then developed several days of markedly decreased oral intake, severe hyperglycemia, probable volume depletion, acute kidney injury, and mild hyperlactatemia. These acute factors occurred in a patient with diabetes, chronic kidney disease, obesity, active tobacco use, and hyperlipidemia, which may collectively increase susceptibility to impaired intestinal perfusion [1-3].

One possible sequence is that persistent erythrocytosis provided a baseline increase in red-cell mass, while acute dehydration caused hemoconcentration and reduced effective circulating volume. Decreased systemic perfusion may then have reduced mesenteric blood flow. Progressive colonic dilation could have further compromised mural perfusion, eventually resulting in extensive ischemic injury and necrosis. Once severe ischemic injury developed, inflammatory and motor dysfunction of the colon may have contributed to the development of toxic megacolon.

This proposed mechanism remains speculative. The available data do not establish that testosterone-induced erythrocytosis caused the ischemic colitis, nor do they demonstrate that dehydration alone was responsible. Similarly, the absence of a demonstrable thrombus does not completely exclude transient thrombosis or microvascular obstruction. Rather, the importance of this case lies in the convergence of several individually modest risk factors that may have produced clinically significant intestinal hypoperfusion in a relatively young patient.

Clinical significance of testosterone-associated erythrocytosis

The case also raises a practical consideration for clinicians managing testosterone therapy. Injectable testosterone is particularly associated with erythrocytosis, and hematocrit monitoring is recommended during treatment [7-9,12]. In patients with additional conditions that may predispose to impaired perfusion, including smoking, diabetes, chronic kidney disease, obstructive sleep apnea, or significant vascular disease, persistent erythrocytosis may warrant closer surveillance even when the hematocrit remains below the threshold generally used to mandate intervention.

At the same time, the literature does not support assuming that every patient with testosterone-associated erythrocytosis is at high risk for thrombosis. Meta-analysis of randomized trials has not demonstrated a definitive increase in venous thromboembolic events attributable to TRT [13]. Therefore, the present case should be viewed as hypothesis-generating rather than evidence of a direct causal relationship between testosterone and ischemic colitis.

Postoperative transition of care

Finally, this case demonstrates that successful treatment of catastrophic bowel ischemia extends beyond operative management. Following subtotal colectomy and ileostomy creation, the patient experienced delays in obtaining appropriate ostomy appliances, skin barriers, barrier rings, and home health services. New ostomy patients may experience peristomal skin complications, leakage, adjustment difficulties, and educational needs related to appliance management, diet, and fluid intake [18,19]. Timely access to ostomy supplies and specialized nursing support is therefore an important component of postoperative care.

In this patient, the need for coordination between colorectal surgery, primary care, home health services, and durable medical equipment providers illustrates the importance of structured transitional care following emergency colorectal surgery. Ensuring that patients leave the hospital with appropriate supplies, education, and follow-up may reduce preventable complications and facilitate recovery.

## Conclusions

This case highlights an unusual presentation of extensive ischemic colitis with toxic megacolon in a relatively young patient without an identified occlusive vascular lesion or conventional thrombophilia. The combination of testosterone-associated persistent erythrocytosis, hereditary hemochromatosis requiring therapeutic phlebotomy, acute volume depletion, severe hyperglycemia, colonic distention, and multiple vascular risk factors provides a plausible multifactorial explanation for impaired colonic perfusion. The case emphasizes that ischemic bowel disease may arise from the interaction of several physiologic insults rather than a single identifiable thrombotic event.
